# Evaluation of Mechanical and Environmental Properties of Engineered Alkali-Activated Green Mortar

**DOI:** 10.3390/ma13184098

**Published:** 2020-09-15

**Authors:** Iman Faridmehr, Ghasan Fahim Huseien, Mohammad Hajmohammadian Baghban

**Affiliations:** 1 Institute of Architecture and Construction, South Ural State University, Lenin Prospect 76, 454080 Chelyabinsk, Russia; s.k.k-co@live.com; 2 Department of Building, School of Design and Environment, National University of Singapore, Singapore 117566, Singapore; eng.gassan@yahoo.com; 3 Department of Manufacturing and Civil Engineering, Norwegian University of Science and Technology (NTNU), 2815 Gjøvik, Norway

**Keywords:** FA, GBFS, POFA, OPC, recycled ceramics, green mortar, alkali-activated mix design, compressive strength, embodied energy, CO_2_ emission

## Abstract

Currently, alkali-activated binders using industrial wastes are considered an environmentally friendly alternative to ordinary Portland cement (OPC), which contributes to addressing the high levels of carbon dioxide (CO_2_) emissions and enlarging embodied energy (EE). Concretes produced from industrial wastes have shown promising environmentally-friendly features with appropriate strength and durability. From this perspective, the compressive strength (CS), CO_2_ emissions, and EE of four industrial powder waste materials, including fly ash (FA), palm oil fly ash (POFA), waste ceramic powder (WCP), and granulated blast-furnace slag (GBFS), were investigated as replacements for OPC. Forty-two engineered alkali-activated mix (AAM) designs with different percentages of the above-mentioned waste materials were experimentally investigated to evaluate the effect of each binder mass percentage on 28-day CS. Additionally, the effects of each industrial powder waste material on SiO_2_, CaO, and Al_2_O_3_ contents were investigated. The results confirm that adding FA to the samples caused a reduction of less than 26% in CS, whereas the replacement of GBFS by different levels of POFA significantly affected the compressive strength of specimens. The results also show that the AAM designs with a high volume FA provided the lowest EE and CO_2_ emission levels compared to other mix designs. Empirical equations were also proposed to estimate the CS, CO_2_ emissions, and EE of AAM designs according to their binder mass compositions.

## 1. Introduction

There is environmental concern worldwide regarding the production of OPC, as it is widely employed as a concrete binder in construction industries. It is commonly accepted that the manufacturing of OPC causes serious pollution issues, including a considerable amount of CO_2_ emissions. It was estimated that around 10 billion metric tons of concrete are produced annually and that the majority of these products contain OPC [1]. Every tonne of OPC creates approximately one tonne of CO_2_; it was estimated that almost 7% of all the CO_2_ emissions produced globally are attributed to OPC production and its equivalent raw material extraction [2]. It was also estimated that the manufacturing of OPC will quadruple over the next three decades, which is expected to lead to extensive environmental, ecological, and economic degradation [3].

To address this concern, reducing the consumption of raw materials and the application of industrial wastes are considered practical solutions for a sustainable and cleaner environment. In general terms, disposal of industrial waste materials is associated with negative ecological impacts, whereas their recycling largely contributes to sustainable design, saving natural resources and preventing the dumping of waste into landfills. The application of industrial wastes with environmentally-friendly (i.e., low energy consumption and carbon dioxide emission) and inexpensive features as a partial or full replacement for OPC in the concrete binder has attracted the attention of many researchers [4,5,6]. Golewski et al. [7] and Panesar et al. [8] found that sustainable design and green concrete concepts could be promoted through the recycling of different wastes, such as fly ash, ceramics, and GBFS as a replacement for natural raw materials. Several researchers also confirmed that the application of alkali-activated mix (AAM) designs using industrial and agriculture waste materials, such as fly ash (FA), recycled ceramics, palm oil fuel ash (POFA), metakaolin (MK), and recycled granulated blast-furnace slag (GBFS), improves the mechanical properties and durability of concrete [9,10,11,12]. They concluded that alkali-activated binders using waste materials provide reliable mechanical properties in harsh environments compared to conventional concrete. Several researchers also examined the mechanical properties of alkali-activated binders, including CS capacity [13,14,15], modulus of elasticity (MoE) [16], permeability and porosity, acid, sulfate resistance [17,18], and autogenous, drying shrinkage, and thermal resistance [19]. It has been shown that, in general, AAC exhibits better bond performance with steel reinforcement and better strength performance after exposure to elevated temperatures than OPCC. The results also indicated that using AAM design also improves the durability and permeability in freeze–thaw resistance, which led to a prolonged life time of the RC building. In addition, the results indicated that AAMs could be used as a base plaster for fire-resistant coatings on combustible materials, forming a barrier layer in order to increase the passive safety of wooden structures in heritage buildings.

GBFS is obtained by quenching molten iron slag (a by-product of iron and steel production) from a blast furnace in water or steam to produce a glassy, granular product that is then dried and ground into a fine powder. GBFS is highly cementitious and high in calcium silicate hydrates (C-S-H), which is a strength-enhancing compound that improves the strength, durability, and appearance of the concrete. The high levels of SiO_2_ and CaO in GBFS provide mechanical properties similar to those of OPC and pozzolan. Accordingly, GBFS has become a popular choice as a replacement for OPC. Previous studies confirmed that including GBFS in alkali-activated binders improves their mechanical properties and resilience along with changing their microstructure [20]. Nevertheless, the relatively high CO_2_ emissions, cost, and embodied energy (EE) demand during preparation, as well as the low durability to sulfuric acid (high CaO content) and fast setting times, restrict the application of GBFS on a large scale in the construction industry [21].

FA, a by-product of burnt coal in thermal power stations, contains a relatively low level of Ca and is among the best industrial wastes for producing an alkali-activated binder due to several unique features, including availability at a large scale in many countries, high levels of SiO_2_ and Al_2_O_3_, and low cost and EE. Additionally, FA-based geopolymer binders display a reliable resilience to heat curing. Nevertheless, the main drawbacks associated with the application of FA are its poor strength when cured in ambient temperature, the high required molarity (UP 10 M) of sodium hydroxide, and high demand for an alkaline solution [22]. Accordingly, there is no benefit of using FA separately to achieve a satisfactory alkali-activated binder. Several studies were conducted to determine a solution to increasing the strength of FA, mainly by incorporating ingredients containing calcium [23]. Generally, calcium has the potential to improve the mechanical properties of alkali-activated binders, where calcium (aluminum) silicate hydrate (C-(A)-S-H) gels are created along with sodium aluminum silicate hydrate (N-A-S-H) [24].

POFA is a by-product mainly produced by diverse agriculture industries in Indonesia, Malaysia, and Thailand, and obtained during the burning of waste materials, such as palm kernel shells, palm oil fiber, and palm oil husks. A survey revealed that the production of POFA in the above-mentioned countries is continuously increasing [25]. POFA has no market value, and its application is normally limited to land filling in lagoons and ponds, which is a major environmental concern. Recent studies classified POFA as a pozzolanic substance that is rich in silica content [26]. Accordingly, such a cheap and abundantly available resource material can be used as a partial substitute for OPC or as an AAM binder to improve strength and durability.

ACIMAC, the association of Italian manufacturers of machinery and equipment for the ceramic industry, published a report indicating that around 13.7 billion m^2^ ceramic tiles were produced in 2018 worldwide [27]. Around 30% of the products in this industry go to waste. Waste ceramics are significantly resistant and durable to harsh environmental conditions. To date, this waste material has mainly been recycled as a filler in tartan floors and gardening. As an effort toward sustainable design, several studies proposed the application of ceramic waste in concrete and mortar and concluded that such wastes significantly improved the workability of fresh mortars due to the excess amount of water absorption that occurred during the geopolymerization process [28,29,30]. Lu et al. [31] found that including fine ceramic aggregates (up to 50%) in a concrete mix design improved the concrete’s durability performance and CS.

Several studies addressed the application of industrial waste materials in alkali-activated mix designs as a sustainable substitute for OPC [32,33,34]. Nevertheless, several issues, such as a reliable method to predict the mechanical behavior and optimum composition of the binder mass, did not receive enough attention, leading to a limit on their mass production. In this study, the 28-day CS of zero-OPC binder with ternary blended alkali-activated mix designs composed of industrial waste materials (FA, GBFS, POFA, and WCP) was investigated using experimental tests and scanning electron microscopy (SEM). Since the AAM designs are affected by the chemical composition of each industrial waste material, the effects of each material replacement on SiO_2_, CaO, and Al_2_O_3_ contents were investigated in detail. The CO_2_ emissions, and EE of all AAM designs with different binder mass constitutions were also investigated using a cradle-to-gate approach. Empirical equations were also proposed in this paper to estimate the CS, CO_2_ emissions, and EE of AAM designs according to their binder mass compositions.

## 2. Experimental Program

### 2.1. Wastes Material Characterization

Based on trial mixes, the optimum values of sodium hydroxide molarity, sodium silicate to sodium hydroxide ratio, alkaline solution to binder ratio, and binder to aggregate ratio were selected and these values were fixed for all cement-free AAM designs. Ternary blended contents using GBFS, FA and POFA, or WCP were examined to determine the influence of calcium oxide on the geopolymerization process. GBFS content as a source of CaO was kept to a minimum of 20% in the replacement process and a maximum of 70%. By using an X-ray fluorescence spectroscopy (XRF) test, the chemical compositions of the studied waste material were determined, as shown in Table 1.

Table 1 reveals that the major compound in FA, WCP, and POFA is SiO_2_ (57.2, 72.6, and 64.2%, respectively), whereas in GBFS, it is CaO (51.8%). Al_2_O_3_, SiO_2_, and CaO are essential oxides throughout the hydration process and production process of the C(A)SH gels. Nevertheless, the low contents of CaO and Al_2_O_3_ in WCP require including materials comprising high quantities of CaO (GBFS) and Al_2_O_3_ (FA) to produce high-performance alkali-activated binders. According to ASTM C618-15 [35], FA and WCP are classified as class F pozzolans as a result of the existence (higher than 70%) of SiO_2_ + Al2O_3_ + Fe_2_O_3_.

To prepare POFA and WCP industrial waste materials powder, the Loss Angelos Abrasion machine that operates with 15 stainless balls each of diameter 50 mm, drum speed in the range of 32–35 revolutions per minute (rpm) had been used. The duration of grinding influences the finesse of particles, which is monitored every one hour. In this research, the percentage of particles was retained at 45 μm.

### 2.2. Scanning Electron Microscope (SEM) Images

**Figure 1:**
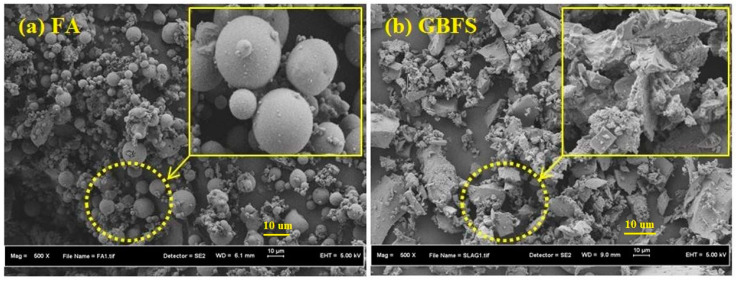
SEM images of (**a**) FA and (**b**) GBFS.

High-Resolution, magnified images in SEM are useful within materials science to test the quality of materials, ensuring that they are fit for purpose and can be used to predict and prevent material failure. SEM analyses were performed using a Hitachi SU8020 microscope with an Energy Dispersive Spectroscopy (EDS). SEM images of FA revealed that it is comprised of regular spherical particles, while GBFS is comprised of irregular angular particles, as shown in Figure 1.

**Figure 2:**
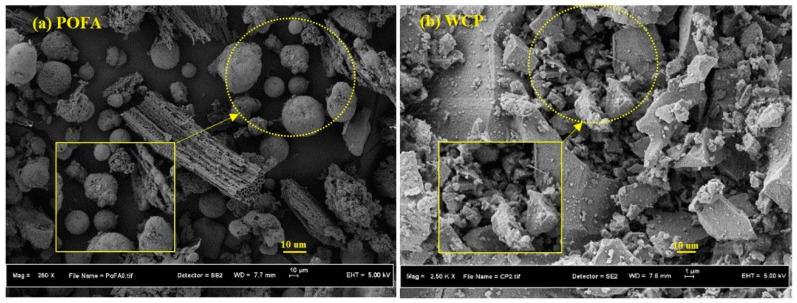
SEM images of (**a**) POFA and (**b**) ceramic waste.

Recycled tile ceramic waste with the same thickness and no glassy coating was collected, resulting in homogeneous waste. The manufacturing process of WCP involved several steps, including (1) crushing ceramic tiles using a jaw crusher, (2) sieving with a 600 μm sieve to remove large particles, and (3) grinding using a Los Angeles abrasion machine for 6 h. To prepare POFA, large particles were first removed using a 600 μm sieve, which were then dried in an oven at 105 °C for 24 h. Subsequently, they were sieved using a 300 μm sieve and ground using a Los Angeles abrasion machine for 6 h to achieve fine particles. The SEM image of POFA clearly revealed the presence of irregular and spherical particles, whereas the SEM image of the ceramic waste indicated it contained irregular and angular particles, as shown in Figure 2.

### 2.3. Design of the Alkali-Activated Mix Designs

Based on trial mixes, the optimum values of sodium hydroxide molarity, sodium silicate to sodium hydroxide ratio, alkaline solution to binder ratio, and binder to fine aggregate ratio were selected as 4 M, 0.75, 0.40, and 1.0, respectively. These values were fixed for all alkali-activated mix designs. Additionally, all mixtures had a standard and fixed proportion of fine and course aggregate. Analytical-grade NaOH (NH, 98% purity) was purchased from QREC (Johor, Malaysia) as an alkali activator to prepare the proposed alkali-activated mortars. An analytical grade sodium silicate solution comprised of SiO_2_ (29.5 wt %), Na_2_O (14.70 wt %), and H_2_O (55.80 wt %) was procured from QREC (Johor, Malaysia). The NaOH pellets were dissolved in water to prepare the alkaline solution of 4 M concentration. The solution was first cooled for 24 h and then added to sodium silicate (NS) solution to obtain an alkaline solution with a SiO_2_ to Na_2_O ratio of 1.02. The ratio of NS to NH was fixed to 0.75 for all the alkaline mixtures. Overall, four distinct AAM designs with ternary blended contents using GBFS, FA, POFA, and WCP were examined to determine the influence of calcium oxide on the geopolymerization process and CS.

#### 2.3.1. High Volume Fly Ash Mix Design

This section describes the effect of GBFS replacement by FA at various levels on the contents of SiO_2_, CaO, and Al_2_O_3_ in AAMs. Three levels of replacement were adopted to evaluate the effect of CaO on the geopolymerization process. At each level, the minimum content of GBFS was kept as low as 20%. At every level, the GBFS was replaced by POFA to evaluate the effect of increasing silica oxide and reducing aluminum oxide on AAM properties. Table 2 displays the effect of GBFS replaced by FA and POFA replaced by GBFS on SiO_2_, CaO, and Al_2_O_3_ contents. CaO content was observed to increase with increasing GBFS content and decreasing FA and POFA contents. CaO content increased from 19.2% to 23.8% and 28.5% with the replacement of FA by GBFS with 30%, 40%, and 50% portions, respectively. SiO_2_ content increased with the increase in POFA content. However, Al_2_O_3_ content decreased with increasing GBFS and POFA contents and decreasing FA content.

#### 2.3.2. High Volume Palm Oil Fuel Ash Mix Design

Nine mixtures were prepared to evaluate the high-volume content of POFA on the AAM properties, as shown in Table 3. The high level of POFA (70%) was reduced to 60% and 50% via the replacement by GBFS and FA. The GBFS content was kept between 20% and 50%. The results revealed that a reduction in the POFA content from 70% to 50% led to reducing the silicate content. The Al_2_O_3_ content increased when the ratio of GBFS to FA increased, while the CaO content significantly declined.

#### 2.3.3. High Volume GBFS Mix Design

To evaluate the effect of high CaO contents on the geopolymerization process and the properties of AAM designs, 15 mixes were synthesized. Three levels of GBFS content were prepared, with 50%, 60%, and 70%. Then, the GBFS was replaced by FA and POFA. Table 4 illustrates the effects of GBFS content on SiO_2_, CaO, and Al_2_O_3_ contents. An increase in the GBFS content from 50% to 60% and 70% was observed to lead to an increase in CaO content from 28.5% to 33.2% and 37.8%, respectively. Additionally, SiO_2_ content increased with the reduction in GBFS content and the increase in POFA content, whereas Al_2_O_3_ content increased with increasing FA content.

#### 2.3.4. High Volume Ceramic Waste Mix Design

The effect of CaO on activated blended content with a high amount of SiO_2_ and Al_2_O_3_ was considered in this mix design. Nine mixtures containing a high amount of ceramic waste were prepared. Three levels of ceramic waste were adopted, including 50%, 60%, and 70%, and replaced with GBFS and FA. The minimum content of GBFS was kept at 20% and the maximum was kept at 50%. The effect of the high volume of WCP and the ratio of GBFS to FA on the contents of SiO_2_, CaO, and Al_2_O_3_ are shown in Table 5. An increase in the content of WCP led to an enhancement of SiO_2_ content. The replacement of WCP by increasing the amount of GBFS led to an increase in the CaO content. Al_2_O_3_ content also increased with increasing FA content.

### 2.4. Test Procedure

For the casting process, the resulting mortar was poured into the molds using the two-layers pouring method. In this process, each layer was subject to a vibration for a period of 15 s in order to eliminate any air pockets within the mixture. Once the casting process was completed, the AAMs were cured for 24 h in an ambient atmosphere (temperature 24 ± 1.5 °C, relative humidity 75%) before the demolding process. The compression strength of all specimens was measured at 28 days, following ASTM C109-109M [36]. A constant loading rate of 2.5 kN/s was applied to all tested specimens and equivalent compressive strengths were recorded automatically based on the size of the specimens. Once the specimens were broken, they were ground to a fine powder for SEM analysis. Scanning electron microscopy (SEM) with sufficient magnification was used to examine the AAMs’ surface morphology. First, the AAM samples were collected from the specimens tested for compressive strength at 28 days of age, and then each sample was sowed on to double cellophane sheets and then attached to a coin. All specimens were coated in advance using a gold sputter coater machine. Significant morphology images were captured immediately after selecting the sufficient image magnifications.

## 3. Results and Discussion

### 3.1. Mechanical Properties

**Figure 3:**
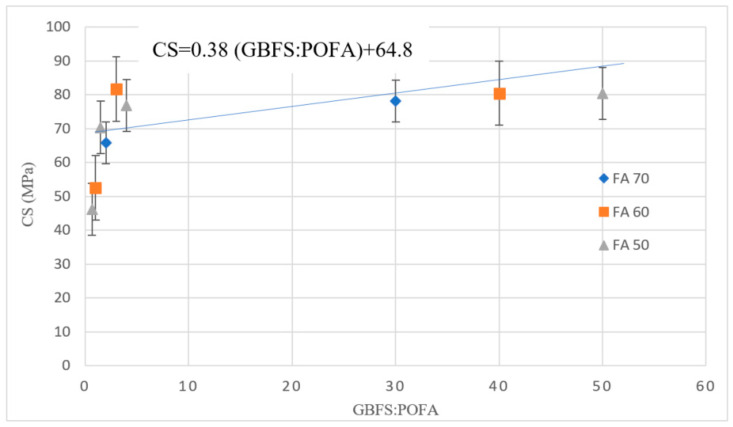
CS of AAM designs with a high volume of FA.

The impacts of AAM designs with different binder mass constituents on CS are presented in Table 2, Table 3, Table 4, Table 5 and discussed in this section. The 28-day CS of the AAM designs with a high-volume FA substitute is presented in Figure 3. The results indicated that the highest compressive strength was achieved by the GBFS to POFA ratio of 4 with 60% FA. The trend line demonstrates that by increasing the ratio of GBFS to POFA, CS increased for all mix designs with different FA percentages.

**Figure 4:**
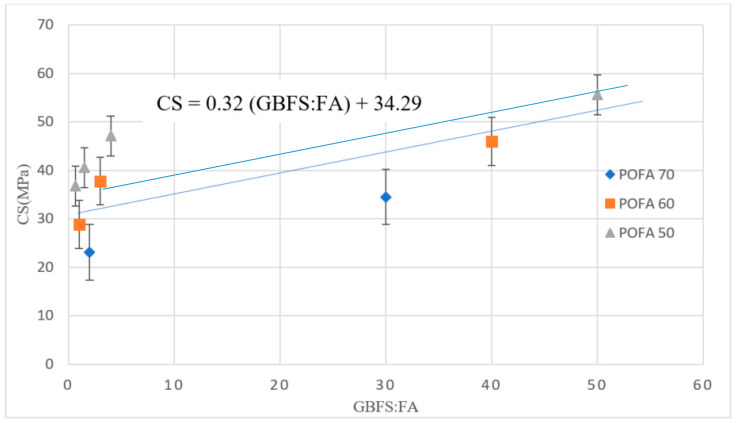
CS of AAM designs with a high volume of POFA.

Figure 4 shows the 28-day CS versus the ratio of GBFS to FA in AAM designs with a high volume POFA. The results confirmed that by increasing POFA, the CS decreased. Additionally, the trend line shows that increasing the ratio of GBFS to FA improved the CS regardless of POFA percentage. The reduction in CS of this mix design containing POFA is ascribed to the delay in the hydration process as well as the slow pozzolanic activity of POFA, which negatively influences the CS.

**Figure 5:**
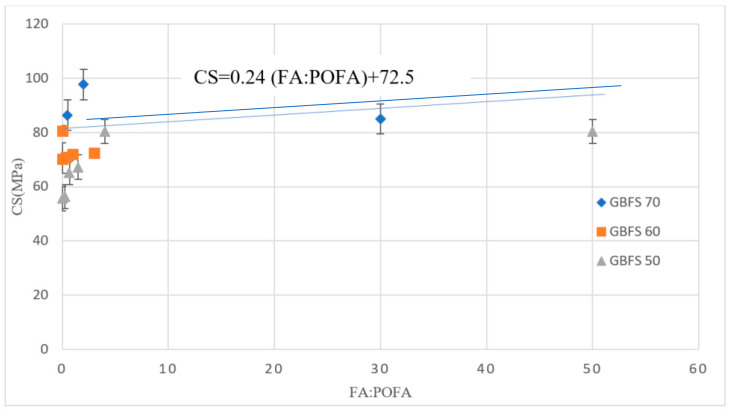
CS of AAM designs with a high volume of GBFS.

Figure 5 shows the 28-day CS versus the ratio of FA to POFA in AAM designs with a high volume of GBFS. It is evident that the ratio of FA to POFA had a minor effect on CS. For an almost identical ratio of FA to POFA, the CS increased by around 53% by increasing the GBFS percentage from 50% to 70%.

**Figure 6:**
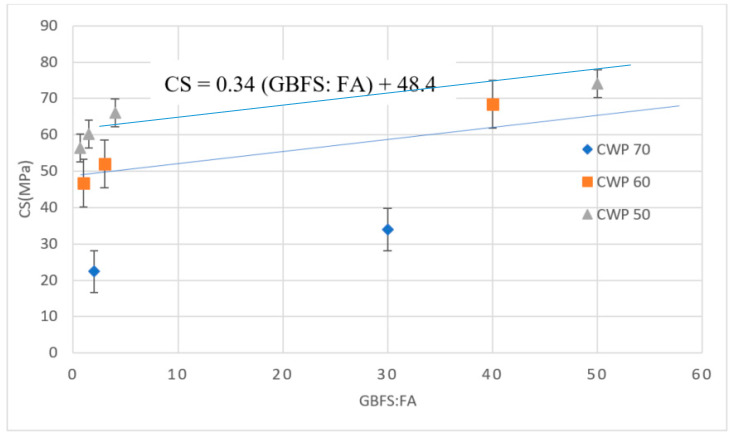
CS of AAM designs with a high volume of WCP.

Figure 6 shows the 28-day CS versus the ratio of GBFS to FA in AAM designs with a high volume of WCP. The results clearly showed that increasing the WCP percentage substantially decreased the CS, where the CS declined from 60 to around 20 MPa by decreasing the WCP from 70% to 50%. Additionally, the results confirmed that CS improves by increasing the ratio of GBFS to FA.

### 3.2. Correlate the Strength with the Chemical Composition of the Mixture

**Figure 7:**
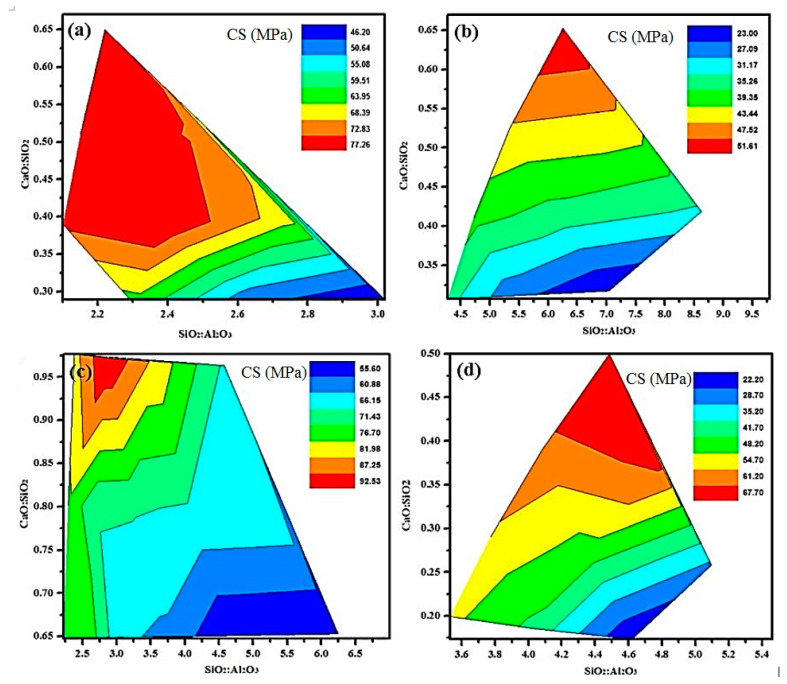
Effect of varying CaO:SiO_2_ and SiO_2_:Al_2_O_3_ on the compressive strength of AAMs containing a high volume of (**a**) FA (**b**) POFA (**c**) GBFS (**d**) WCP.

Figure 7 shows the effects of SiO_2_:Al_2_O_3_ and CaO:SiO_2_ on 28-days CS of ternary blended AAMs. Depending on the percentages of each industrial waste material in ternary blended AAMs, the following conclusion can be drawn:

i. In AAMs with high volume FA, the increment in the SiO_2_ to Al_2_O_3_ ratio resulted in the reaction of Al_2_O_3_ content in the earlier stages. Therefore, the gradual increment of the SiO_2_ content in further stages provided more silicate for condensation and reaction between the silicate species and this caused the dominance of oligomeric silicates. The domination of SiO_2_ content reduced the rate of condensation resulting in gradual hardening of the AAMs (Figure 7a).
ii. In AAMs with high volume POFA, the increase in the POFA to GBFS ratio of the AAMs delayed the ultimate CS (Figure 7b). The developed strength was inversely correlated to the silicate to aluminium ratio, which was recorded to be an optimum of 55.6 MPa for a silicate to aluminium ratio of 6.5.
iii. In AAMs with high volume GBFS, the 28-days CS was highest (97 MPa) for calcium to silicate ratio of 0.97. A ratio of silicate to aluminium of 2.75 and 3.25 reduced the 28-days CS to 86.4 and 85.1 MPa, respectively (Figure 7c).
iv. In AAMs with high volume WCP, it was observed that the compressive strength was enhanced with the decrease in SiO_2_:Al_2_O_3_ and the highest 28-days CS (67 MPa) was recorded with calcium to silicate ratio higher than 0.40 (Figure 7d).

Overall, it is evident that an increase in the percentage content of CaO and Al_2_O_3_ could enhance the CS of the mortar following the above-mentioned mechanism.

### 3.3. Evaluation of SEM Results

**Figure 8:**
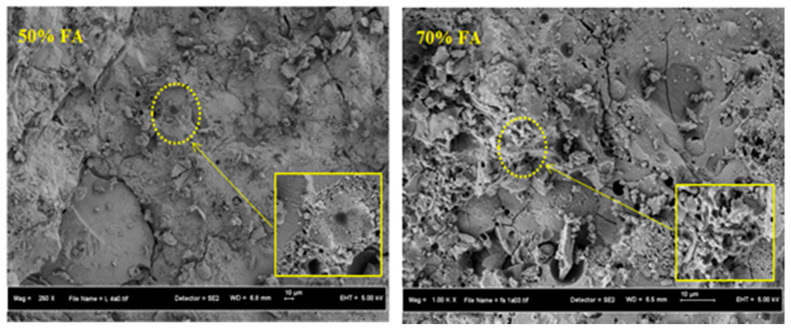
SEM images of AAM designs with different FA contents.

Figure 8 shows the SEM images of the AAM designs with 50% and 70% FA contents after 28 days of curing. The results clearly showed that the specimen with 50% FA possessed less non-reactive particles and micro-cracks compared to 70% FA. Our interpretation of this particular issue is that increasing FA leads to a decrease in CS, as shown in Table 2. It is anticipated that cracks and pores will increase on a larger scale when increasing the FA percentage in AAM designs. The previous literature has also indicated that a reduction in the CS with an increase in FA content was ascribed to the decrease in CaO and increase in SiO_2_ content in the mortar’s matrix [37,38]. Furthermore, increasing the FA content could generate a low CaO to SiO_2_ ratio, leading to the formation of a smaller quantity of C-(A)-S-H gel in the mixtures containing up to 70% FA compared to the mixtures with over 30% addition of FA. It has well known that the increasing content of aluminosilicate materials, such as FA, POFA, and CWP in the alkali-activated matrix led to a rise in the quantity of non-reacted quartz (SiO_2_) which causes a poor morphology structure and high porosity. The performance of AAMs’ strength was adversely influenced by this and showed lower strength values.

**Figure 9:**
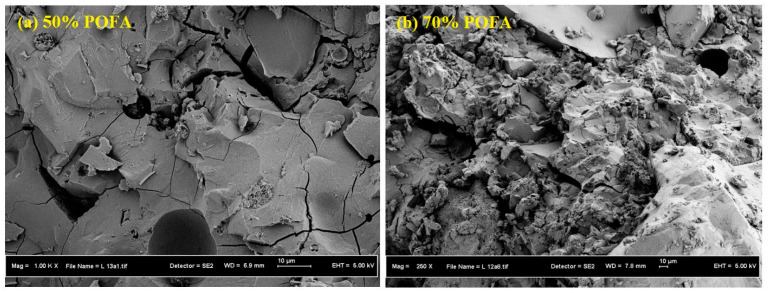
SEM images of AAM mix designs with (**a**) 50% and (**b**) 70% POFA contents.

Figure 9 presents the SEM images of the AAM designs for different POFA contents of 50% and 70%. The results confirmed that at a lower POFA content, the specimen was free from cracks. Increasing the POFA content increased the number of unreacted particles and led to a poor binder structure. The results also indicated that in design mixes with a high POFA content, a large amount of silica remained unreacted, leading to a poor alkali-activated binder structure. This issue is also confirmed in Table 3, where the AAM designs with a high content of POFA led to the lowest CS.

**Figure 10:**
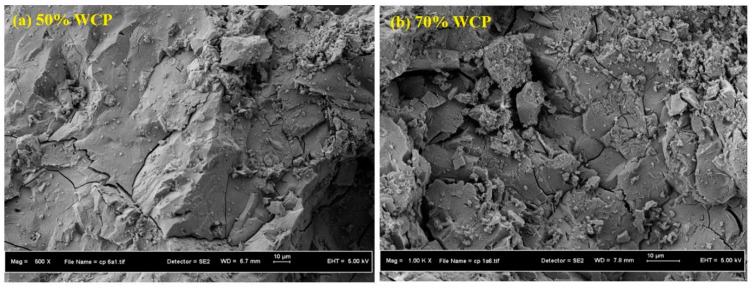
SEM images of AAM designs with (**a**) 50% and (**b**) 70% ceramic contents.

To gain insight into the waste ceramic powder inclusion with different percentages in the alkali-activated mix designs, the SEM images were investigated, as shown in Figure 10. The result indicated the creation of a larger amount of crystalline Ca(OH)_2_ in hexagonal plate-like structures in alkali-activated mix designs with 60% and 70% ceramic contents. This can be explained by the hydration process of an excess amount of CaO in the GBFS and FA, which produces an elevated amount of Ca(OH)_2_ crystals. When GBFS is mixed with FA, it shapes a hydrated binding cement paste (HCP) of calcium silicate hydrate (C-S-H). The formation of a massive amount of C-S-H crystals in alkali-activated mix designs with 50% ceramic content made it denser, which is attributed to the pozzolanic reaction of SiO_2_ with Ca(OH)_2_ during the hydration process. Subsequently, such dense microstructures contribute to enhancing the CS of the mix designs.

### 3.4. Sustainable Properties

**Figure 11:**
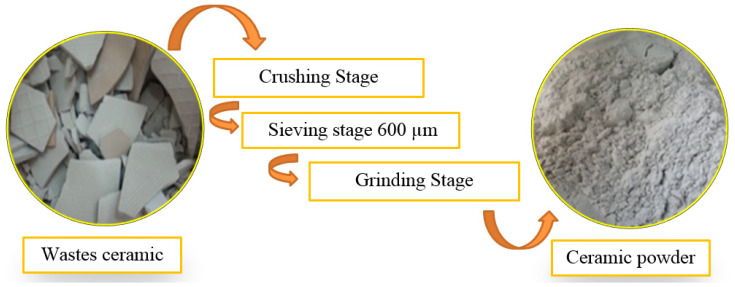
Cradle-to-gate process to produce ceramic powder from waste ceramics.

The energy consumption for the transformation from a raw material to a refined material is defined as the embodied energy (EE). The EE comprises of energy for collecting (i.e., mining process, grinding process, sieving process) and refining (a group of chemical engineering unit processes and unit operations that refine certain materials or convert raw material into products of value). Energy consumption in these two stages is recognized to be the fundamental source of the embodied energies of building materials. Extra energy is required for machines, transportation, labor and facilities. In this research, a cradle-to-gate EE was used to define the energy required for resource extraction (cradle) to the factory gate (i.e., before it is transported to the consumer). An example of a cradle-to-gate process for WCP is shown in Figure 11.

The cradle-to-gate EE of each AAM design with different binder mass constituents can be calculated using the following equation.

(1) $$EE_{i}=E_{extraction,\;i}+E_{manufacture,\;i}$$

(2) $$EE_{AAM}=\overset{i}{\underset{1}{\sum }}EE_{i}m_{i}$$

where $EE_{i}$ and $EE_{AAM}$ are the embodied energies (MJ) of waste materials i and AAM designs i, respectively. $m_{i}$ is the mass of waste material i.

It is evident that, upon the application of industrial waste materials, the EE would significantly decrease. Meanwhile, building energy consumption is responsible for approximately 45% of the CO_2_ emissions in the atmosphere, which contributes to the greenhouse effect. For instance, emissions of CO_2_ from fossil fuel combustion, with contributions from cement manufacture, have been responsible for more than 75% of the increase in atmospheric CO_2_ concentrations since pre-industrial times [39]. The cradle-to-gate CO_2_ emission of each AAM design with different binder mass constituents can be calculated using the following equation.

(3) $$CO_{2,i}=E_{extraction,\;i}+E_{manufacture,\;i}$$

(4) $$CO_{2,\;AAM}=\overset{i}{\underset{1}{\sum }}CO_{2,i}m_{i}$$

where $CO_{2,i}$ and $CO_{2,\;AAM}$ are the CO_2_ emission (kg CO_2_/kg) of waste materials i and AAM designs i, respectively.

The EE of each employed industrial waste material and the relevant CO_2_ emissions are summarized in Table 6. Although the EE of each waste material was calculated by the authors, the accuracy of the estimated values was evaluated by referring to the available guidelines—i.e., the Inventory of Carbon and Energy (ICE) [40]. The results show that OPC required a massive amount of energy for the manufacturing process compared to waste materials, which led to an increase in EE and the release of greenhouse gases.

Table 7 shows the calculated EE and CO_2_ emissions for all AAM designs composed of industrial waste materials. Table 7 confirms that the average EE and CO_2_ emissions of all studied AAM designs are 1.5 MJ/kg and 0.084 kgCO_2_/kg, which are lower than those of OPC. It is also clear that the AAM designs using high FA contents provided the lowest EE and CO_2_ emissions compared to other design mixes.

## 4. Developing an Empirical Equation to Predict Compressive Strength

In this section, using a multi linear regression (MLR), an empirical equations are proposed to estimate the CS, EE, and CO_2_ emissions based on the proportions of each industrial waste used in the binder mass, as shown in Equations (5) to (7).

(5) CS = 20.31+0.397FA+0.886GBFS−0.227POFA+0CWP

(6) EE=1.579 − 1.406 FA + 0.8 GBFS − 0.466 CWP +0.2POFA

(7) $$CO_{2}\;emission=0.059-0.047\;FA\;+0.093\;GBFS\;-0.014\;CWP\;+\;0.012PFOA$$

**Figure 12:**
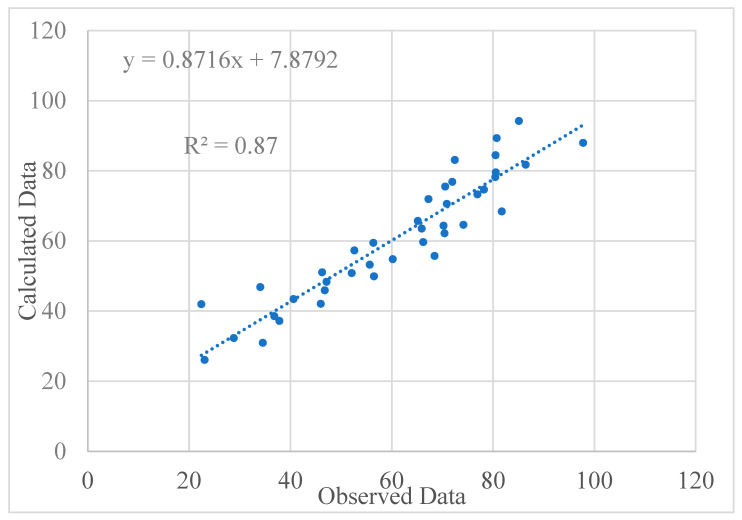
Results of the linear regression empirical equation to estimate the CS parameter.

These equations were developed based on the proportions of each industrial waste used in the binder mass. Figure 12 shows the scatter graph that provided the relationship between the test and calculated data using an empirical equation for estimating the CS. The average and standard deviation of the proposed empirical equation are 58.5 MPa and 19.6, respectively, compared to 61.3 MPa and 18.7, as calculated by the test results; this indicates the adequacy of the proposed empirical equation. The coefficient of determination (*R*^2^), which determines the overall change in the dependent variables calculated by the linear regression equation, Equation (5), was 0.8715, which is relatively acceptable, providing good confidence in the relationship.

**Figure 13:**
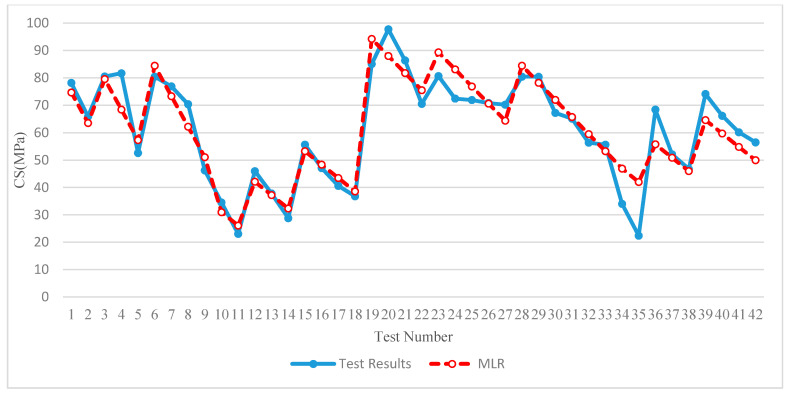
Comparison between observational and computational methods.

Figure 13 shows the comparison between observational (test) and computational (MLR) methods of all 42 specimens for estimating the CS. It is clear that except for some specimens, the MLR approach properly estimated the CS.

**Figure 14:**
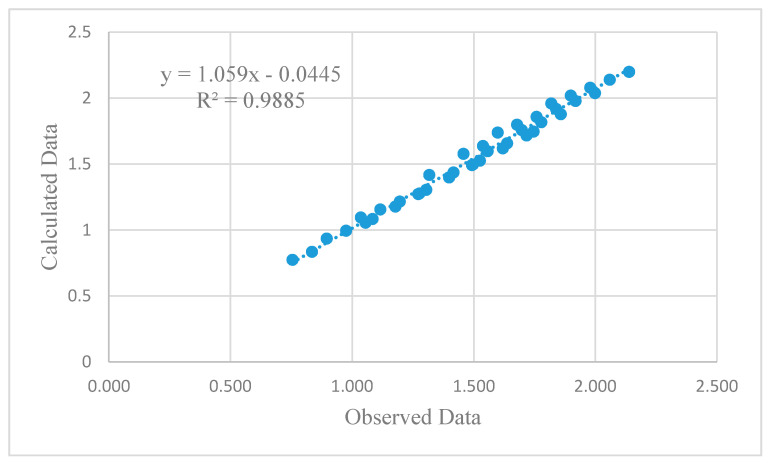
Results of the linear regression empirical equation to estimate the EE parameter.

**Figure 15:**
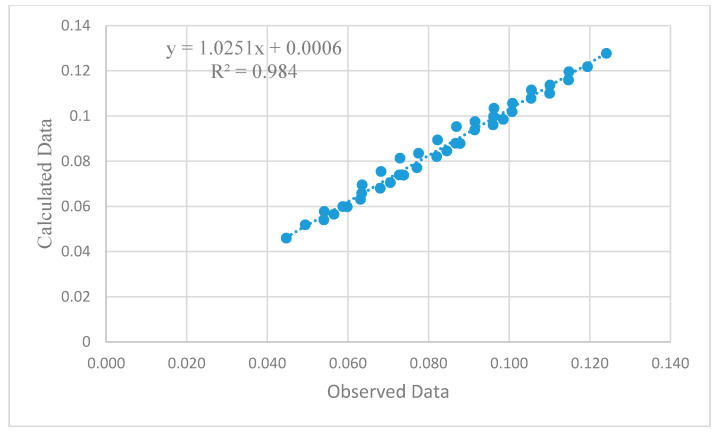
Results of the linear regression empirical equation to estimate the $CO_{2}$ emission parameter.

Figure 14 and Figure 15 show the scatter graph that provided the relationship between the test and calculated data, estimated by an empirical Equations (6) and (7), for estimating the EE and CO_2_ emission, respectively. The coefficient of determination (R^2^) is estimated as 0.98 for both EE and CO_2_ emissions, indicating the adequacy of the proposed equations.

## 5. Conclusions

In this study, the 28-day CS of zero OPC mortar with ternary blended AAM designs composed of industrial waste materials (FA, GBFS, POFA, and WCP) were investigated. The results confirmed that the CS of these environmentally friendly AAM designs were satisfactory, with an average of 61.3 MPa. According to the test results from different ternary blended AAM designs and relevant contents of constituents, the following conclusions were reached:

1. The X-ray fluorescence spectroscopy (XRF) results confirmed that the ratio of GBFS to FA significantly controls the SiO_2_, CaO, and Al_2_O_3_ contents in ternary blended AAM designs.
2. AAM designs with a high volume of GBFS provided the highest 28-day CS, were in these ternary blended mixes. The ratio of FA to POFA had a minor effect on CS; however, increasing the GBFS percentage substantially improved the CS.
3. The CS in all AAM designs declined as a result of increasing the FA content. SEM images also confirmed that the AAM designs using 50% FA possessed less non-reactive particles and micro-cracks compared with 70% FA.
4. The CS in AAM designs with a high volume of WCP and FA was ranked the lowest among all AAM designs—at around 20 MPa. SEM images also confirmed that the formation of a large amount of C-S-H crystals in AAM designs with a low content of WCP increased the density, which was attributed to the pozzolanic reaction of SiO_2_ with Ca(OH)_2_ during the hydration process.
5. The industrial waste materials had significantly lower EE and CO_2_ emissions compared to OPC. The AAM designs that contained a high FA content provided the lowest EE and CO_2_ emissions compared to other design mixes.

## Figures and Tables

**Table 1:** Physical properties and chemical composition of studied waste materials.

Material	GBFS	FA	POFA	WCP
Physical characteristics				
Specific gravity	2.9	2.2	1.96	2.6
Medium particle size (μm)	12.8	10	8.2	35
Chemical composition (% by mass)				
SiO_2_	30.8	57.20	64.20	72.6
Al_2_O_3_	10.9	28.81	4.25	12.6
Fe_2_O_3_	0.64	3.67	3.13	0.56
CaO	51.8	5.16	10.20	0.02
MgO	4.57	1.48	5.90	0.99
K_2_O	0.36	0.94	8.64	0.03
Na_2_O	0.45	0.08	0.10	13.5
SO_3_	0.06	0.10	0.09	0.01
Loss on ignition (LOI)	0.22	0.12	1.73	0.13

**Table 2:** Contents of constituents in the high volume of FA mix design.

No.	Binder (Mass %)			SiO_2_:Al_2_O_3_	CaO:SiO_2_	CaO:Al_2_O_3_
	FA	GBFS	POFA			
1	70	30	0	2.10	0.39	0.82
2		20	10	2.31	0.28	0.66
3	60	40	0	2.15	0.51	1.10
4		30	10	2.38	0.39	0.94
5		20	20	2.62	0.29	0.76
6	50	50	0	2.22	0.65	1.43
7		40	10	2.47	0.51	1.26
8		30	20	2.74	0.40	1.08
9		20	30	3.02	0.29	0.89

**Table 3:** Contents of constituents in high volume POFA mix design.

No.	Binder (Mass %)			SiO_2_:Al_2_O_3_	CaO:SiO_2_	CaO:Al_2_O_3_
	POFA	GBFS	FA			
1	70	30	0	8.63	0.42	3.61
2		20	10	7.04	0.32	2.23
3	60	40	0	7.32	0.53	3.86
4		30	10	6.13	0.41	2.54
5		20	20	5.33	0.31	1.66
6	50	50	0	6.25	0.65	4.08
7		40	10	5.34	0.52	2.80
8		30	20	4.72	0.41	1.94
9		20	30	4.27	0.31	1.31

**Table 4:** Contents of constituents in high volume GBFS mix design.

No.	Binder (Mass %)					
	GBFS	FA	POFA	SiO_2_:Al_2_O_3_	CaO:SiO_2_	CaO:Al_2_O_3_
1	70	30	0	2.38	0.97	2.32
2		20	10	2.85	0.97	2.77
3		10	20	3.53	0.96	3.41
4		0	30	4.57	0.96	4.41
5	60	40	0	2.29	0.80	1.83
6		30	10	2.69	0.80	2.15
7		20	20	3.25	0.79	2.59
8		10	30	4.06	0.79	3.23
9		0	40	5.35	0.79	4.25
10	50	50	0	2.22	0.65	1.43
11		40	10	2.57	0.65	1.66
12		30	20	3.04	0.65	1.97
13		20	30	3.68	0.65	2.39
14		10	40	4.65	0.65	3.03
15		0	50	6.25	0.65	4.07

**Table 5:** Contents of constituents in high volume WCP.

No.	Binder (Mass %)					
	WCP	FA	GBFS	SiO_2_:Al_2_O_3_	CaO:SiO_2_	CaO:Al_2_O_3_
1	70	0	30	5.09	0.26	1.31
2		10	20	4.62	0.17	0.79
3	60	0	40	4.79	0.37	1.77
4		10	30	4.35	0.27	1.19
5		20	20	4.01	0.18	0.74
6	50	0	50	4.48	0.50	2.24
7		10	40	4.08	0.39	1.59
8		20	30	3.77	0.29	1.09
9		30	20	3.53	0.20	0.70

**Table 6:** EE and CO_2_ emission of employed industrial waste material and OPC.

Materials	CO_2_ Emission (kg CO_2_/kg)	EE (MJ/kg)
WCP	0.04	1.11
POFA	0.06	1.58
FA	0.01	0.17
GBFS	0.15	2.37
OPC	0.90	5.13

**Table 7:** Calculated EE and CO_2_ emission for all the studied AAM designs.

AAM Designs	Binder Constitution (Composed of Industrial Waste Materials)				Sustainable and Mechanical Features		
	FA	GBFS	WCP	POFA	EE (MJ/kg)	CO_2_ Emission (kgCO_2_/kg)	28-Days CS (MPa)
1	0.70	0.30	0.00	0.00	0.83	0.05	78.18
2	0.70	0.20	0.00	0.10	0.75	0.04	65.89
3	0.60	0.40	0.00	0.00	1.05	0.06	80.51
4	0.60	0.30	0.00	0.10	0.97	0.05	81.70
5	0.60	0.20	0.00	0.20	0.89	0.04	52.60
6	0.50	0.50	0.00	0.00	1.27	0.08	80.46
7	0.50	0.40	0.00	0.10	1.19	0.07	76.90
8	0.50	0.30	0.00	0.20	1.11	0.06	70.40
9	0.50	0.20	0.00	0.30	1.03	0.05	46.24
10	0.00	0.30	0.00	0.70	1.81	0.08	34.53
11	0.10	0.20	0.00	0.70	1.59	0.07	23.04
12	0.00	0.40	0.00	0.60	1.89	0.09	45.96
13	0.10	0.30	0.00	0.60	1.67	0.08	37.80
14	0.20	0.20	0.00	0.60	1.45	0.06	28.80
15	0.00	0.50	0.00	0.50	1.97	0.10	55.64
16	0.10	0.40	0.00	0.50	1.75	0.09	47.10
17	0.20	0.30	0.00	0.50	1.53	0.07	40.60
18	0.30	0.20	0.00	0.50	1.31	0.06	36.80
19	0.30	0.70	0.00	0.00	1.71	0.11	85.09
20	0.20	0.70	0.00	0.10	1.85	0.11	97.75
21	0.10	0.70	0.00	0.20	1.99	0.11	86.40
22	0.00	0.70	0.00	0.30	2.13	0.12	70.53
23	0.40	0.60	0.00	0.00	1.49	0.09	80.68
24	0.30	0.60	0.00	0.10	1.63	0.10	72.44
25	0.20	0.60	0.00	0.20	1.77	0.10	71.93
26	0.10	0.60	0.00	0.30	1.91	0.11	70.84
27	0.00	0.60	0.00	0.40	2.05	0.11	70.22
28	0.50	0.50	0.00	0.00	1.27	0.08	80.46
29	0.40	0.50	0.00	0.10	1.41	0.08	80.43
30	0.30	0.50	0.00	0.20	1.55	0.09	67.22
31	0.20	0.50	0.00	0.30	1.69	0.09	65.14
32	0.10	0.50	0.00	0.40	1.83	0.10	56.34
33	0.00	0.50	0.00	0.50	1.97	0.10	55.64
34	0.00	0.30	0.70	0.00	1.49	0.07	34.02
35	0.10	0.20	0.70	0.00	1.27	0.06	22.40
36	0.00	0.40	0.60	0.00	1.61	0.08	68.44
37	0.10	0.30	0.60	0.00	1.39	0.07	52.08
38	0.20	0.20	0.60	0.00	1.17	0.06	46.76
39	0.00	0.50	0.50	0.00	1.74	0.09	74.12
40	0.10	0.40	0.50	0.00	1.52	0.08	66.19
41	0.20	0.30	0.50	0.00	1.30	0.07	60.17
42	0.30	0.20	0.50	0.00	1.08	0.05	56.47
Average					1.50	0.08	61.30
STDEV					0.36	0.02	18.70

## References

[1] Jacobsen S., Jahren P. Binding of CO_2_ by carbonation of Norwegian OPC concrete. Proceedings of the CANMET/ACI International Conference on Sustainability and Concrete Technology.

[2] Turner L.K., Collins F.G. (2013). Carbon dioxide equivalent (CO_2_-e) emissions: A comparison between geopolymer and OPC cement concrete. Constr. Build. Mater..

[3] Scrivener K.L., John V.M., Gartner E.M., UN Environment (2018). Eco-efficient cements: Potential economically viable solutions for a low-CO_2_ cement-based materials industry. Cem. Concr. Res..

[4] Katare V.D., Madurwar M.V., Raut S. (2020). Agro-Industrial Waste as a Cementitious Binder for Sustainable Concrete: An Overview. Sustainable Waste Management: Policies and Case Studies.

[5] Al-Kutti W., Nasir M., Johari M.A.M., Islam A.S., Manda A.A., Blaisi N.I. (2018). An overview and experimental study on hybrid binders containing date palm ash, fly ash, OPC and activator composites. Constr. Build. Mater..

[6] Almalkawi A.T., Balchandra A., Soroushian P. (2019). Potential of using industrial wastes for production of geopolymer binder as green construction materials. Constr. Build. Mater..

[7] Golewski G.L. (2018). Green concrete composite incorporating fly ash with high strength and fracture toughness. J. Clean. Prod..

[8] Panesar D.K., Seto K.E., Churchill C.J. (2017). Impact of the selection of functional unit on the life cycle assessment of green concrete. Int. J. Life Cycle Assess..

[9] Zawawi M.N.A.A., Muthusamy K., Majeed A.P.A., Musa R.M., Budiea A.M.A. (2020). Mechanical properties of oil palm waste lightweight aggregate concrete with fly ash as fine aggregate replacement. J. Build. Eng..

[10] Li F., Liu L., Yang Z., Li S. (2020). Physical and mechanical properties and micro characteristics of fly ash-based geopolymer paste incorporated with waste Granulated Blast Furnace Slag (GBFS) and functionalized Multi-Walled Carbon Nanotubes (MWCNTs). J. Hazard. Mater..

[11] Anderson D.J., Smith S.T., Au F.T. (2016). Mechanical properties of concrete utilising waste ceramic as coarse aggregate. Constr. Build. Mater..

[12] Ranjbar N., Behnia A., Alsubari B., Birgani P.M., Jumaat M.Z. (2016). Durability and mechanical properties of self-compacting concrete incorporating palm oil fuel ash. J. Clean. Prod..

[13] Kastiukas G., Zhou X., Castro-Gomes J. (2017). Preparation conditions for the synthesis of alkali-activated binders using tungsten mining waste. J. Mater. Civ. Eng..

[14] Nikoo M., Torabian Moghadam F., Sadowski Ł. (2015). Prediction of concrete compressive strength by evolutionary artificial neural networks. Adv. Mater. Sci. Eng..

[15] Nikoo M., Zarfam P., Sayahpour H. (2015). Determination of compressive strength of concrete using Self Organization Feature Map (SOFM). Eng. Comput..

[16] Zhang P., Wang K., Li Q., Wang J., Ling Y. (2020). Fabrication and Engineering Properties of Concretes Based on Geopolymers/Alkali-activated Binders—A Review. J. Clean. Prod..

[17] Zhang W., Yao X., Yang T., Zhang Z. (2018). The degradation mechanisms of alkali-activated fly ash/slag blend cements exposed to sulphuric acid. Constr. Build. Mater..

[18] Huseien G.F., Sam A.R.M., Shah K.W., Mirza J., Tahir M.M. (2019). Evaluation of alkali-activated mortars containing high volume waste ceramic powder and fly ash replacing GBFS. Constr. Build. Mater..

[19] Son H., Park S.M., Seo J.H., Lee H.K. (2019). Effect of CaSO_4_ incorporation on pore structure and drying shrinkage of alkali-activated binders. Materials.

[20] Huseien G.F., Shah K.W. (2020). Durability and life cycle evaluation of self-compacting concrete containing fly ash as GBFS replacement with alkali activation. Constr. Build. Mater..

[21] Kumar V., Kumar A., Prasad B. (2019). Mechanical behavior of non-silicate based alkali-activated ground granulated blast furnace slag. Constr. Build. Mater..

[22] Golewski G.L. (2020). Energy Savings Associated with the Use of Fly Ash and Nanoadditives in the Cement Composition. Energies.

[23] Meesala C.R., Verma N.K., Kumar S. (2020). Critical review on fly-ash based geopolymer concrete. Struct. Concr..

[24] Singh N., Kumar P., Goyal P. (2019). Reviewing the behaviour of high volume fly ash based self compacting concrete. J. Build. Eng..

[25] Jokhio G., Hamada H.M., Humada A.M., Gul Y., Abu-Tair A. (2020). Environmental benefits of incorporating palm oil fuel ash in cement concrete and cement mortar. E3S Web Conf..

[26] Tang W.L., Lee H.S., Vimonsatit V., Htut T., Singh J.K., Wan Hassan W.N.F., Ismail M.A., Seikh A.H., Alharthi N. (2019). Optimization of micro and nano palm oil fuel ash to determine the carbonation resistance of the concrete in accelerated condition. Materials.

[27] Ferrari A.M., Volpi L., Pini M., Siligardi C., García-Muiña F.E., Settembre-Blundo D. (2019). Building a sustainability benchmarking framework of ceramic tiles based on life cycle sustainability assessment (LCSA). Resources.

[28] Samadi M., Huseien G.F., Mohammadhosseini H., Lee H.S., Lim N.H.A.S., Tahir M.M., Alyousef R. (2020). Waste ceramic as low cost and eco-friendly materials in the production of sustainable mortars. J. Clean. Prod..

[29] Mohit M., Sharifi Y. (2019). Thermal and microstructure properties of cement mortar containing ceramic waste powder as alternative cementitious materials. Constr. Build. Mater..

[30] Goldaran R., Turer A. (2020). Application of acoustic emission for damage classification and assessment of corrosion in pre-stressed concrete pipes. Measurement.

[31] Lu G., Fan Z., Sun Z., Liu P., Leng Z., Wang D., Oeser M. (2020). Improving the polishing resistance of cement mortar by using recycled ceramic. Resour. Conserv. Recycl..

[32] Awoyera P., Adesina A., Sivakrishna A., Gobinath R., Kumar K.R., Srinivas A. (2020). Alkali activated binders: Challenges and opportunities. Mater. Today Proc..

[33] Adesina A. (2019). Properties of alkali activated slag concrete incorporating waste materials as aggregate: A review. Materials Science Forum.

[34] Goldaran R., Lofollahi-Yaghin M.A., Aminfar M.H., Turer A. (2017). Investigation of attenuation and acoustic wave propagation path caused by corrosion for reliability assessment of prestressed pipe monitoring using Acoustic Emission technique. Modares Mech. Eng..

[35] (2015). C618-15 Standard Specification for Coal Fly Ash and Raw or Calcined Natural Pozzolan for Use in Concrete.

[36] (2013). Standard Test Method for Compressive Strength of Hydraulic Cement Mortars (using 2-in. or [50-mm] Cube Specimens).

[37] Huseien G.F., Shah K.W. (2020). Performance evaluation of alkali-activated mortars containing industrial wastes as surface repair materials. J. Build. Eng..

[38] Hwang C.-L., Yehualaw M.D., Vo D.H., Huynh T.P., Largo A. (2019). Performance evaluation of alkali activated mortar containing high volume of waste brick powder blended with ground granulated blast furnace slag cured at ambient temperature. Constr. Build. Mater..

[39] Pachauri R.K., Reisinger A. (2007). IPCC Fourth Assessment Report.

[40] Hammond G., Jones C. (2011). Embodied Carbon: The Inventory of Carbon and Energy (ICE).

